# Serum Metabolomic Analysis of Chronic Drug-Induced Liver Injury With or Without Cirrhosis

**DOI:** 10.3389/fmed.2021.640799

**Published:** 2021-03-29

**Authors:** Shuai-shuai Chen, Ying Huang, Yu-ming Guo, Shan-shan Li, Zhuo Shi, Ming Niu, Zheng-sheng Zou, Xiao-he Xiao, Jia-bo Wang

**Affiliations:** ^1^School of Pharmacy, Chengdu University of Traditional Chinese Medicine, Chengdu, China; ^2^Department of Liver Diseases, The Fifth Medical Center of Chinese People's Liberation Army General Hospital, Beijing, China; ^3^Department of Poisoning Treatment, The Fifth Medical Center of Chinese People's Liberation Army General Hospital, Beijing, China; ^4^School of Traditional Chinese Medicine, Capital Medical University, Beijing, China

**Keywords:** biomarker, chronic drug-induced liver injury, cirrhosis, fingerprint, metabolomics, signature

## Abstract

**Background:** Chronic drug-induced liver injury (DILI) occurs in up to 20% of all DILI patients. It presents a chronic pattern with persistent or relapsed episodes and may even progress to cirrhosis. However, its underlying development mechanism is poorly understood.

**Aims:** To find serum metabolite signatures of chronic DILI with or without cirrhosis, and to elucidate the underlying mechanism.

**Methods:** Untargeted metabolomics coupled with pattern recognition approaches were used to profile and extract metabolite signatures from 83 chronic DILI patients, including 58 non-cirrhosis (NC) cases, 14 compensated cirrhosis (CC) cases, and 11 decompensated cirrhosis (DC) cases.

**Results:** Of the 269 annotated metabolites associated with chronic DILI, metabolic fingerprints associated with cirrhosis (including 30 metabolites) and decompensation (including 25 metabolites), were identified. There was a significantly positive correlation between cirrhosis-associated fingerprint (eigenmetabolite) and the aspartate aminotransferase-to-platelet ratio index (APRI) (*r* = 0.315, *P* = 0.003). The efficacy of cirrhosis-associated eigenmetabolite coupled with APRI to identify cirrhosis from non-cirrhosis patients was significantly better than APRI alone [area under the curve (AUC) value 0.914 vs. 0.573]. The decompensation-associated fingerprint (eigenmetabolite) can effectively identify the compensation and decompensation periods (AUC value 0.954). The results of the metabolic fingerprint pathway analysis suggest that the blocked tricarboxylic acid cycle (TCA cycle) and intermediary metabolism, excessive accumulation of bile acids, and perturbed amino acid metabolism are potential mechanisms in the occurrence and development of chronic DILI-associated cirrhosis.

**Conclusions:** The metabolomic fingerprints characterize different stages of chronic DILI progression and deepen the understanding of the metabolic reprogramming mechanism of chronic DILI progression to cirrhosis.

## Introduction

DILI is one of the most common and serious adverse reactions to drugs (1–5), and an important cause of clinically acute liver injury and failure (2, 6, 7). Clinically, most patients with DILI recover after drug withdrawal. However, around 8 ~ 20% of patients progress to chronicity (2, 8–10). According to the Spanish DILI registry study (10), 8% (25/298) of DILI patients had unresolved liver injury over 1 year, and among these chronic DILI patients, 64% (16/25) did not resolve in the first 3 years. More importantly, 44% (7/16) of these chronic DILI patients who underwent liver biopsies had cirrhosis and 6% (1/16) manifested fibrosis. Collectively, a high proportion of patients with chronic DILI may progress into cirrhosis. However, the mechanism of chronic DILI progression is still unclear.

Metabolomics is the study of small molecular metabolites. It improves our understanding of the pathophysiological evolution of diseases and greatly promotes the diagnosis, treatment, and prognosis of diseases (11). Metabolic fingerprints are a cluster composed of a series of serum metabolites that highly correlate with diseases (12). They have great significance in revealing the pathophysiological mechanism of diseases and in searching for biomarkers. Metabolomic methods have been used successfully to identify biomarkers associated with DILI. Previous studies reported that metabolomic biomarkers predicted the occurrence of DILI (13, 14) and distinguished between DILI and autoimmune hepatitis (15). Xie et al. (16) reported that changes in metabolomic biomarkers, such as glycochenodeoxycholic acid, phosphatidylcholine, and two metabolomic pathways (primary bile acid biosynthesis and alpha-linolenic acid metabolism) are closely related to the severity of DILI. These studies suggest the robustness and capability of untargeted metabolomics in screening for DILI-related biomarkers. However, the metabolomics of chronic DILI progression has not been studied.

Thus, we conducted untargeted metabolomic analysis of 83 chronic DILI patients (hepatitis, compensated cirrhosis, decompensated cirrhosis) and compared three groups of serum metabolomics characteristics, to establish the metabolic fingerprint of chronic DILI-related cirrhosis, including its potential biomarkers and mechanism of occurrence and development.

## Materials and Methods

### Study Design

Patients from the Fifth Medical Center of Chinese PLA General Hospital from 2015 to 2017, were enrolled. The enrolled patients were diagnosed according to DILI guidelines (2) and liver cirrhosis guidelines (17), with no age and sex restriction. A total of 83 hospitalized chronic DILI patients, including non-cirrhosis (NC, *n* = 58), compensated cirrhosis (CC, *n* = 14), and decompensated cirrhosis (DC, *n* = 11) groups were enrolled. In addition, 10 serum samples of healthy subjects (HS) were collected. This research scheme was approved by the Ethics Committee of the hospital based on the ethical principles of the Declaration of Helsinki. Also, written informed consent was obtained from all patients. Based on the utilization of untargeted metabolomics, we obtained metabolic entities (metabolome profile) with significant difference (*P* < 0.05) between cirrhosis (CC + DC) and NC groups. The differentially abundant metabolites from metabolome profiles were deemed to be cirrhosis-associated metabolome features. Similarly, the differentially abundant metabolites for comparisons between DC and CC groups were considered as decompensation-associated metabolome features. The metabolic fingerprint (a cluster of metabolites) was screened using a hierarchical cluster and heat map analysis. We mainly used unsupervised principal component analysis (PCA) for fingerprint metabolites and formed one eigenmetabolite, which is defined as the first principal component (12).

### Sample Preparation and LC/MS Conditions

Serum samples from the three groups were processed using an organic solvent precipitation method, according to our previous work (14). Ten microliter from each sample was mixed and then prepared as the quality control samples (QC). Agilent ZORBAX 300 SB-C18 column (2.1 × 100 mm, 1.8 μm, Agilent Technologies, USA) was used and the temperature was maintained at 30°C. The temperature of the sample was maintained at 4°C. An optimum mobile phase was composed of water (A, containing 0.1% formic acid and 5% acetonitrile) and acetonitrile (B, containing 0.1% formic acid). The flow rate was maintained at 0.3 mL/min with gradient elution conditions set as follows: 0–1 min, A (95%); 1–9 min, B (5% to 40%); 9–19 min, B (40% to 90%); 19–21 min, B (90% to 100%); 21–25 min, B (100%). The sample injection volume was kept at 4 μL.

Mass spectrometry (MS) evaluations were conducted on an Agilent 6550 iFunnel Q-TOF LC/MS equipped with electron spray ionization (ESI). MS data in both positive and negative modes were acquired based on optimal parameters, including a mass range from m/z 50–1,200; capillary voltage of 2.2 kV (negative) and 2.5 kV (positive); cone voltage of 40 V; ion source temperature of 130°C; desolvation temperature of 350°C; cone airflow of 50 L/h; and desolvation airflow of 800 L/h.

### Statistical Analysis

Mass hunter Profinder software was used for raw data pre-processing. The online analysis tool MetaboAnalyst 4.0 (https://www.metaboanalyst.ca/) was used for data filtering and normalization. The clinical data of patients was expressed as a median (p25, p75) or numerical value. Differences between groups were analyzed by the non-parametric test in SPSS software program (version 25.0, Chicago, IL, USA) and reported as statistically significant if the *P* < 0.05. PCA and orthogonal partial least square discrimination analysis (OPLS-DA) were carried out using the SIMCA-P software (version 14.1, Umetrics AB, Umea, Sweden). The MetaboAnalyst website was used to acquire AUC and the associated *P*-values of differential metabolites. The human metabolome database (HMDB, http://www.hmdb.ca) and Kyoto encyclopedia of genes and genomes (https://www.kegg.jp) were used to construct an interactive network for the metabolic fingerprint and associated metabolic pathway.

## Results

### Demographic and Clinical Parameters of the Study Population

The demographic and clinical parameters of the three groups of study population are listed in Table 1, and the causative agents of DILI patients are presented in Table 2. Patients from NC, CC, and DC groups with no significant differences in the mean ages, with female predominance (*n* = 66, 79.52%) were included in this study. Comparisons between CC and NC groups were conducted and the levels of cholinesterase, platelet, albumin/globulin ratio, and pre-albumin were found to be significantly lower in CC than NC, and the reverse was true for total bile acid. Patients in the DC group showed significantly higher values of direct bilirubin, international normalized ratio, and immunoglobulin M, but significantly lower values of albumin, albumin/globulin ratio, and pre-albumin, compared to the CC group. These results suggest that a gradual disturbance of liver functions occurred during anabolism and metabolism in chronic DILI advancement.

**Table 1:** Clinical characteristics of chronic DILI patients in NC, CC, and DC groups.

**Characteristics**	**NC (*n* = 58)**	**CC (*n* = 14)**	**DC (*n* = 11)**	***P*****-value**	
				**NC *vs*. CC**	**CC *vs*. DC**
Age/year	48.0 (39.5, 55.5)	50.0 (46.0, 52.2)	59.0 (46.0, 67.0)	0.490	0.153
Sex (male/female)	11/47	4/10	2/9	–	–
Alanine aminotransferase/U·L^−1^	35.0 (18.0, 94.0)	43.0 (17.8, 132.2)	32.0 (15.0, 41.0)	0.733	0.066
Aspartate aminotransferase/U·L^−1^	49.5 (24.5, 117.2)	39.5 (24.5, 137.5)	53.0 (33.0, 112.0)	0.876	0.641
Alkaline phosphatase/U·L^−1^	96.0 (77.2, 126.2)	120.0 (69.5, 195.5)	137.0 (103.0, 211.0)	0.252	0.443
Total bilirubin/μmo·L^−1^	12.5 (8.4, 19.4)	14.9 (9.7, 35.1)	32.0 (20.4, 70.2)	0.289	0.055
Direct bilirubin/μmo·L^−1^	4.2 (3.2, 10.4)	5.3 (4.1, 18.6)	14.8 (7.1, 49.4)	0.261	0.025
Total bile acid/μmo·L^−1^	11.0 (5.0, 18.0)	25.0 (6.5, 40.5)	36.0 (20.0, 72.0)	0.040	0.188
International normalized ratio/IU	0.9 (0.8, 1.0)	1.0 (0.9, 1.1)	1.1 (1.0, 1.2)	0.039	0.021
γ-glutamyl transpeptidase/U·L^−1^	57.5 (22.0, 148.5)	36.0 (14.7, 110.5)	86.0 (40.0, 140.0)	0.351	0.171
Cholinesterase/U·L^−1^	6,212.0 (5,383.7, 7,330.0)	5,407.0 (4,653.7, 5,902.0)	3,426.0 (1,489.0, 5,910.0)	0.010	0.063
Total cholesterol/mmo·L^−1^	4.3 (3.4, 5.1)	3.8 (3.1, 4.8)	4.5 (2.8, 4.9)	0.224	0.477
Triglyceride/mmo·L^−1^	1.2 (1.0, 1.8)	1.36 (1.17, 1.5)	0.9 (0.8, 1.4)	0.654	0.095
Creatinine/μmol·L^−1^	61.0 (56.0, 68.2)	63.5 (58.2, 69.5)	67.0 (54.0, 80.0)	0.555	0.381
Immunoglobulin A/g·L^−1^	2.2 (1.6, 2.9)	2.6 (1.8, 3.5)	3.7 (2.2, 5.8)	0.164	0.192
Immunoglobulin G/g·L^−1^	12.6 (10.5, 14.4)	14.4 (11.7, 17.1)	16.8 (13.6, 22.8)	0.191	0.099
Immunoglobulin M/g·L^−1^	1.1 (0.8, 1.6)	1.2 (0.6, 1.7)	2.1 (1.3, 2.3)	0.976	0.034
White blood cell/10^9^·L^−1^	5.0 (3.9, 6.3)	5.8 (3.2, 7.2)	4.8 (3.6, 7.8)	0.842	1.000
Platelet/10^9^·L^−1^	204.0 (168.5, 235.0)	154.0 (109.2, 223.5)	121.0 (103.0, 182.0)	0.044	0.352
Albumin/g·L^−1^	38.0 (35.0, 39.0)	36.5 (34.7, 39.0)	27.0 (26.0, 34.0)	0.410	0.002
Globulin/g·L^−1^	27.0 (24.0, 31.0)	32.0 (24.7, 34.0)	33.0 (27.0, 34.0)	0.127	0.297
Albumin/Globulin ratio	1.4 (1.2, 1.5)	1.2 (1.1, 1.4)	0.9 (0.7, 1.0)	0.041	0.003
Prealbumin/mg·L^−1^	156.0 (128.5, 206.5)	104.5 (77.5, 164.8)	81.0 (36.0, 121.0)	0.032	0.046
**Roussel Uclaf causality assessment method score**					
highly probable (>8)	7	3	2		
probable (6 ~ 8)	35	7	6		
possible (3 ~ 5)	16	4	3		

*Data are median (p25, p75) or numerical value. P-values for comparisons were carried out by nonparametric tests*.

**Table 2:** The causative agents of enrolled DILI patients.

**Classification**	**Ingredient**	***n***
Herbal and traditional medicine (H/TM)	TM preparations (ingredient unknown)	19
	Propolis	6
	*Bupleurum chinense* DC	4
	*Dictamnius dasycarpus* Turcz.	2
	*Polygonum multiflorum* Thunb.	2
	*Corydalis yanhusuo* W. T. Wang	2
	*Agrimonia pilosa* Ledeb.	2
	*Rubia cordifolia* L.	1
	*Tripterygium wilfordii* Hook. f.	1
	*Polygonum multiflorum* Thunb./*Psoralea corylifolia* L.	1
	*Bupleurum chinense* DC./*Corydalis yanhusuo* W. T. Wang	1
Chemicals drugs (CDs)	Ibuprofen	6
	Amlodipine	3
	Omeprazole	3
	Amoxicillin	2
	Nitrofurantoin	2
	Phenobarbital	2
	Valproate	2
	Sulfasalazine	2
	Simvastatin	2
	Enalapril	2
	Pioglitazone	2
	Loratadine	1
	Nimesulide	1
	Nifedipine	1
	Methotrexate	1
	Metronidazole	1
	Valsartan	1
	Metronidazole/Amoxicillin	1
	Levofloxacin/Sertraline	1
	Enalapril/Simvastatin	1
	Sertraline/Valproate	1
	Isoniazid/Pyrazinamide/Rifampicin	1
Combined use of H/TM and CDs	*Bupleurum chinense* DC./Simvastatin	1
	*Bupleurum chinense* DC./Amoxicillin-Clavulanate	1
	*Polygonum multiflorum* Thunb./Amoxicillin	1

### Serum Metabolic Profiles in Chronic DILI Evolution

**Figure 1:**
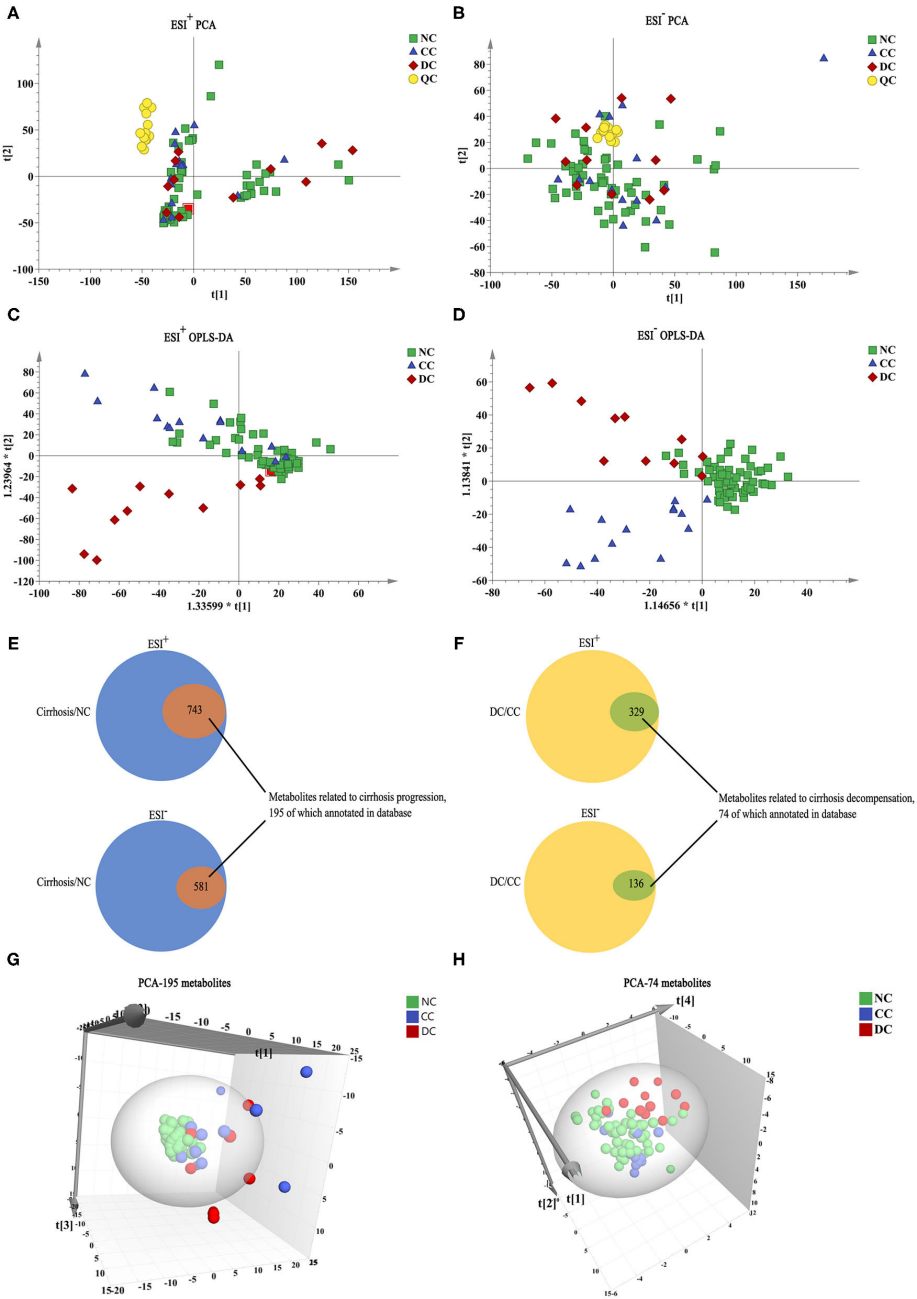
Metabolome profile for chronic DILI with or without cirrhosis. **(A–D)** PCA, and OPLS-DA model analysis of all variables among NC group (*n* = 58, green square), CC group (*n* = 14, blue triangle), DC group (*n* = 11, red diamond), and QC (*n* = 14, yellow circle). **(E,F)** Venn diagram, the inner section of diagram has a set of variables associated with cirrhosis and decompensation, 195 and 74 metabolites annotated for metabolome feature cirrhosis and decompensation, respectively. **(G,H)** PCA 3-dimensional scatter plot of cirrhosis (CC + DC) *vs*. NC with 195 cirrhosis-related metabolites and DC *vs*. CC with 74 decompensated-related metabolites.

Significant changes in global metabolic profiles were observed in different stages of chronic DILI under both ESI^+^ and ESI^−^ modes (Figures 1A–D). This suggests that sera metabolome changed while progressing from NC to CC and DC. To explore the relationship between the evolving stages of chronic DILI and serum metabolite levels, we first screened a set of variables with significant differences (*P* < 0.05) between cirrhosis (CC + DC) and NC, as well as DC *vs*. CC. Of the 1,324 metabolites (including positive and negative ion modes) differentiating between cirrhosis and NC groups, 195 metabolites in the database were annotated (Figure 1E). This indicates that metabolites are related to cirrhosis irrespective of the status of the compensation. Similarly, a total of 465 metabolites (74 were annotated in the database) were identified and differentiated between the DC and CC groups, indicating that these findings are associated with decompensation of cirrhosis (Figure 1F). Then, the three-dimensional PCA scatter plot revealed good separation of 195 cirrhosis-related metabolites between cirrhosis and NC groups (Figure 1G) and 74 decompensation-related metabolites between CC and DC groups (Figure 1H). In summary, the metabolome profiles associated with cirrhosis of chronic DILI and those related to decompensation, were preliminarily found to have potential roles in the identification of cirrhosis or decompensation, respectively.

### Identification of Cirrhosis-Related Metabolic Fingerprint

**Figure 2:**
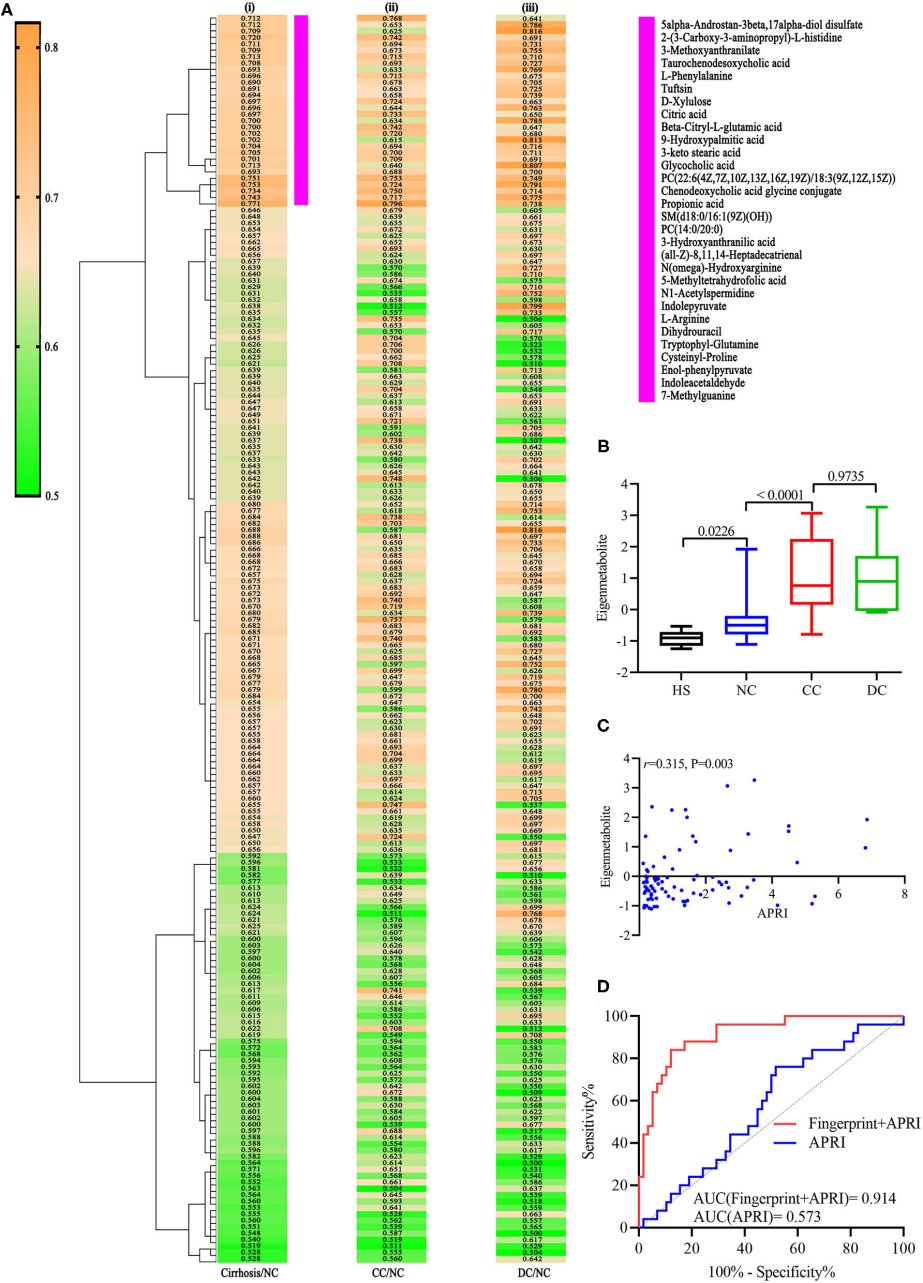
Identification of cirrhosis-related metabolic fingerprint. **(A**i**)** Hierarchical cluster analysis of AUC and P differentiation between cirrhosis and non-cirrhosis groups. **(A**ii**)** Corresponding metabolites in differentiating between DC and NC groups. **(A**iii**)** Indicates corresponding metabolites in differentiating between DC and NC groups. **(B)** shows increasing trend of eigenmetabolite across different stages of chronic DILI, from HS to DC. **(C)** Indicates significantly positive correlation relationship between cirrhosis-associated eigenmetabolite and APRI score. **(D)** is ROC curve analysis for metabolic fingerprint (eigenmetabolite) and APRI score, in discriminating cirrhosis from non-cirrhotic patients with chronic DILI.

According to literature, the AUC and *P*-values of 195 annotated metabolites related to cirrhosis were calculated, to identify a unique metabolomic fingerprint of chronic DILI-related liver cirrhosis (12). The AUC and associated *P*-values were performed using a hierarchical cluster and heat map analysis. Next, we identified the highest relevant cluster with the top 30 metabolites, which had a highly significant association with cirrhosis (vertical violet bar, Figure 2Ai). The AUC and *P*-values for this cluster of metabolites are shown in Supplementary Table 1. Interestingly, we observed that this cluster was effective in distinguishing between CC and NC groups (Figure 2Aii), as well as DC *vs*. NC groups (Figure 2Aiii). We then performed an eigenmetabolite (12) analysis of 30-metabolite clusters by reducing the dimensions and observed that the eigenmetabolite from HS, NC, and CC to DC has been on the rise, whereas no statistically significant differences between CC and DC groups were observed (Figure 2B). Meanwhile, we also found that eigenmetabolite positively correlated with the fibrosis stage in liver biopsy (Supplementary Figure 1A). These results suggest that a single metabolomic fingerprint (30 metabolites) was associated with chronic DILI-related liver cirrhosis, regardless of the compensatory status.

### Comparison of Cirrhosis-Associated Metabolic Fingerprint and APRI in the Identification of Cirrhosis and Non-Cirrhosis

Further, we observed that 30 metabolites consisting of eigenmetabolite are associated with APRI (*r* = 0.315, *P* = 0.003, Figure 2C). We also found that cirrhosis-associated metabolic fingerprints coupled with APRI had better ability for differentiating patients with cirrhosis from those without cirrhosis as compared to APRI alone (AUC values 0.914 *vs*. 0.573, Figure 2D). Collectively, these findings indicate that the 30-metabolite cluster can be used as a cirrhosis-associated fingerprint to assist the identification of cirrhosis and non-cirrhosis cases.

### Identification of Decompensated Cirrhosis-Related Metabolic Fingerprint

**Figure 3:**
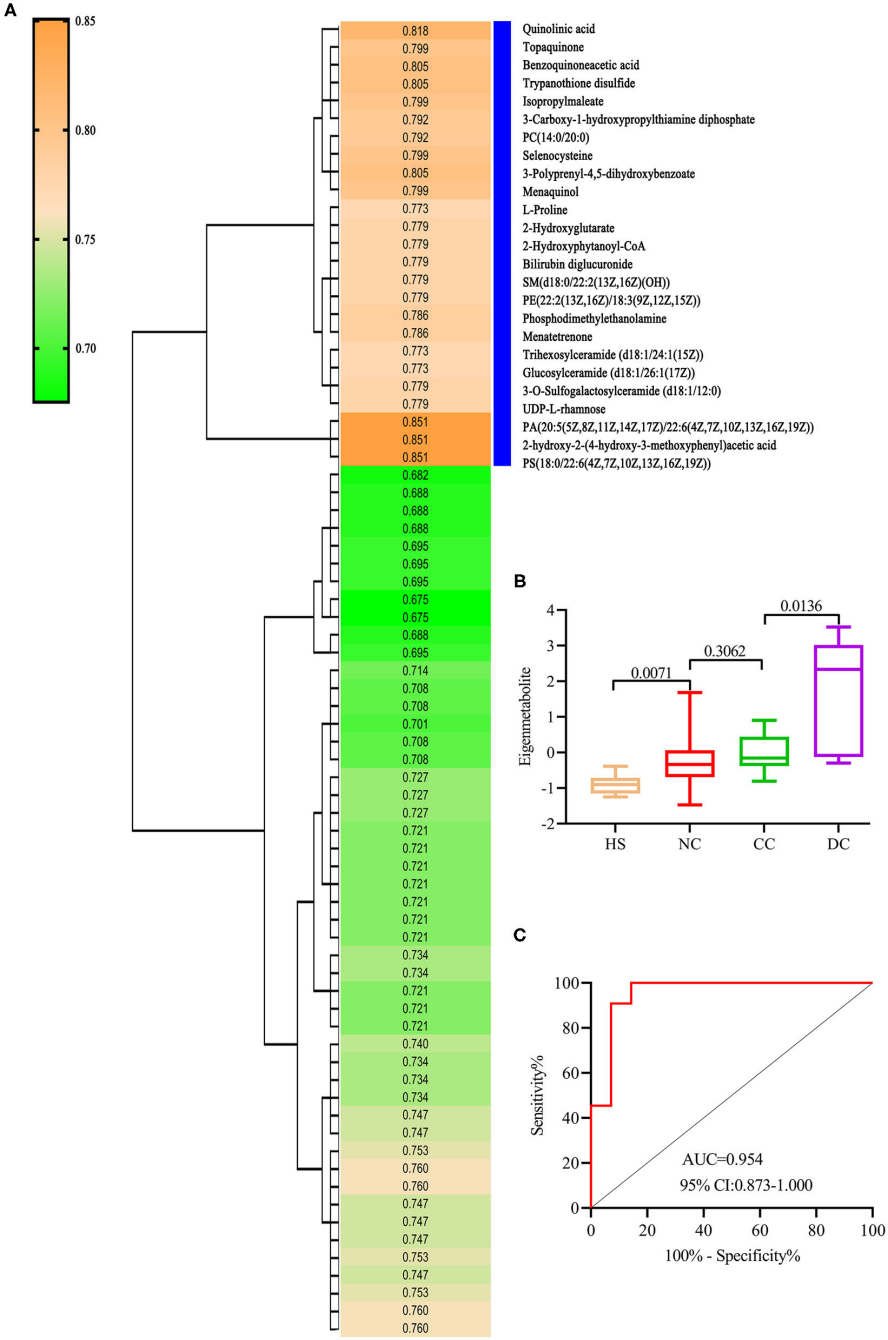
Identification of decompensation-related metabolic fingerprint. **(A)** is a hierarchical cluster analysis of AUC and P differentiation between DC and CC groups. **(B)** shows an increasing trend of eigenmetabolite from HS, NC, and CC to DC. **(C)** ROC analysis for fingerprint (eigenmetabolite) to differentiate between DC and CC groups.

We refined a unique metabolic fingerprint associated with decompensation among the 74 decompensation-related metabolites, in the same way. We then defined the highest relevant cluster with the top 25 metabolites (vertical blue bar, Figure 3A), which were highly associated with DC patients. The resulting AUC and *P*-values of this cluster are presented in Supplementary Table 2. Similarly, we computed an eigenmetabolite for 25-metabolite clusters. Significantly increased eigenmetabolite levels were found in DC patients (Figure 3B) and patients with fibrosis stage 2–3 in liver biopsy (Supplementary Figure 1B). The 25 metabolites consisted of eigenmetabolite that could effectively discriminate patients with decompensation from those in the compensation stage (AUC value 0.954, Figure 3C). In summary, these findings suggest that the identified decompensation-associated metabolic fingerprint could well-discern the decompensation and compensation status in chronic DILI cases.

### Metabolic Fingerprints Have Potential Pathophysiological Significance in Patients of Chronic DILI With Cirrhosis

**Figure 4:**
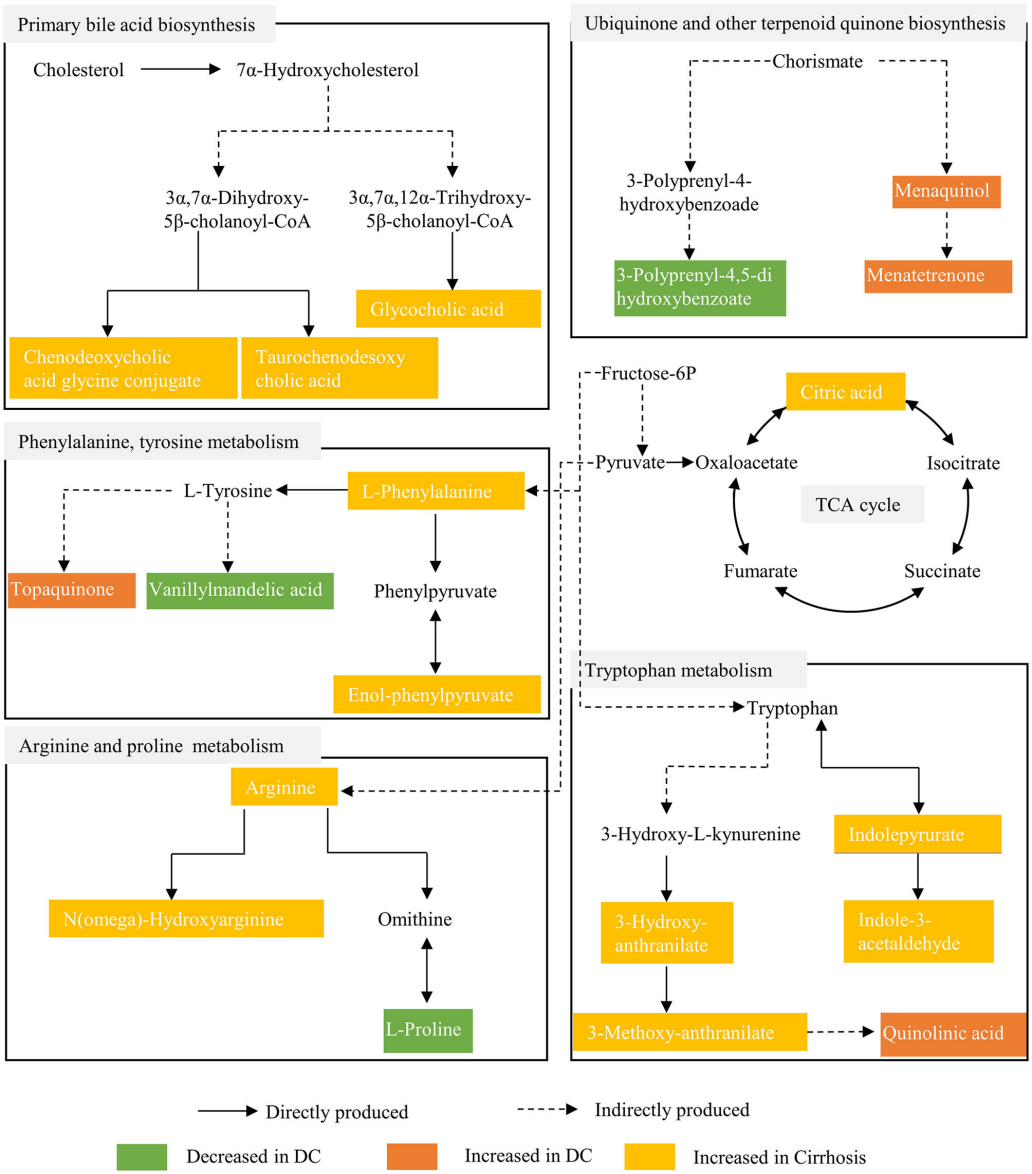
Metabolic pathway alterations involved in chronic DILI with cirrhosis.

To understand the evolution of chronic DILI, two metabolic fingerprints and the associated pathways that form an interactive network were identified, as shown in the schematic diagram (Figure 4). The characteristic metabolic fingerprints of chronic DILI-related cirrhosis are mainly concentrated as described below: phenylalanine and tyrosine metabolism, tryptophan metabolism, arginine and proline metabolism, TCA cycle, ubiquinone and other terpenoid-quinone biosynthesis, and bile acid biosynthesis.

## Discussion

This study characterizes the metabonomic profile of chronic DILI with or without cirrhosis, and reveals the metabolic fingerprints by using untargeted metabolomics and pattern recognition analysis. There are two crucial metabolic fingerprints of chronic DILI without correlation on age and sex (Supplementary Figures 1C,D). One is composed of 30 metabolites reflecting the cirrhosis associated characteristics (with no relationship with decompensation), the other one is decompensation-related metabolic fingerprint, composed of 25 metabolites (focus on the development from the compensated stage to the decompensated stage). Specifically, the metabolic fingerprints of chronic DILI-associated cirrhosis are mainly related to perturbed amino acid metabolism, blocked TCA cycle, intermediate metabolism, and accumulated bile acids.

Glucose metabolism of the liver is disarray in cirrhosis and depends more on proteolysis to provide energy (18–20). Two metabolic fingerprints reveal the metabolites in the amino acid metabolic pathway (phenylalanine metabolism, tryptophan metabolism, and arginine metabolism), with the elevation of proteolysis markers (tryptophyl-glutamine and cysteinyl-proline) in patients with chronic DILI-related cirrhosis (Supplementary Table 3). L-phenylalanine and enol-phenylpyruvate are metabolites of phenylalanine metabolism. L-phenylalanine has been reported to be positively correlated with imaging findings in liver injury patients and progression in liver cirrhosis patients (21, 22). A recent study reported that L-phenylalanine and phenylpyruvate via glycolysis inhibition affect energy metabolism of hepatocytes (23). Indolepyruvate, indole-acetaldehyde, 3-hydroxy-anthranilate, 3-methoxy-anthranilate, and quinolinic acid are metabolites of tryptophan metabolism. Tryptophan and its metabolites affect the immune and nervous system functions of patients (24). Additionally, 3-hydroxy-anthranilate has been reported to significantly provoke impairment of energy metabolism by inhibiting the activities of complexes I and II of the respiratory chain (25). Moreover, there is increased accumulation of quinolinic acid, which reflects not only the degree of liver dysfunction (26), but also inhibits the oxidative phosphorylation involved in cell energy metabolism (27). Arginine and n(omega)- hydroxyarginine are metabolites of arginine metabolism. Under cirrhosis conditions, cytokines and endotoxin may perpetuate arginine metabolism and nitric oxide (NO) generation (28). Arginine has been reported to diminish succinate dehydrogenase and complex II activities through NO formation (29). These findings suggest that energy requirements may lead to increased proteolysis to produce amino acids and their metabolites, which in turn, result in mitochondrial damage and energy metabolism dysfunction.

The disturbed TCA cycle and intermediate metabolism are some additional metabolic features of chronic DILI-associated cirrhosis. We observed a significant increase in the metabolites of TCA cycle (e.g., citric acid and 3-carboxy-1-hydroxypropylthiae diphosphate) in patients with chronic DILI-related cirrhosis (Supplementary Table 3). Citric acid is often revealed as a marker of hepatotoxicity (30). The TCA cycle mainly occurs in mitochondria is the core of intermediate metabolism (31). Blocked TCA cycle causes suppression of both fatty acid oxidation and carbohydrate catabolism, and leads to marked decrease of energy production from nutrients. We also observed some disorders of intermediate metabolites associated with coenzyme Q (e.g., 3-Polyprenyl-4,5-dihydroxybenzoate, menaquinol, menatetrenone), which plays a key role in the electron transport chain and in turn interferes with the electron transport chain and mitochondrial metabolism (32). Disturbed lipid metabolism has been reported as the potential biomarkers of hepatocellular, mixed, and cholestatic-type DILI (33), such as Polygonum Multiflorum-induced liver injury (34). Metabolism disorders of these lipids (e.g., phosphatidylcholine, phosphatidylethanolamine, phosphatidylserine, sphingomyelin, and ceramide; Supplementary Table 3) could result in hepatocyte dysfunction and liver disease progression (35, 36). The deposition of many long-chain fatty acids (e.g., 3-keto stearic acid, all-Z-8,11,14-Heptadecatrienal, 9-hydroxypalmitic acid; Supplementary Table 3) may indicate the inhibition of beta - oxidation in patients with chronic DILI-related cirrhosis, resulting in abnormal fat deposition and energy metabolism (37, 38). These findings therefore indicate that blocked TCA cycle and intermediary metabolism may be contributors to mitochondrial damage.

As main synthesis and metabolism in liver, bile acid is seriously affected after liver disease. We found a significant increase in conjugated bile acids (e.g., glycocholic acid, taurochenodesoxycholic acid, and chenodeoxycholic acid glycine conjugate) in patients with chronic DILI-associated cirrhosis. The excessive accumulation of chenodeoxycholic acid glycine conjugate and taurochenodesoxycholic acid promote apoptosis of liver cells and liver failure (39, 40). These abnormal accumulations of bile acids may not only be biomarkers for chronic DILI-associated cirrhosis, but also involved in the development of the disease. Inhibiting accumulation of toxic bile acids in the liver may be beneficial to the clinical treatment of chronic DILI, while there are very few studies in this area, and hence it is worth exploring. Collectively, these findings indicate mitochondrial injury, lipid accumulation, and bile acid accumulation may be as mechanism implicated in DILI, which is consistent with the literature (41).

Our analysis provides novel information to further our understanding of the pathological changes of several metabolic pathways, based on serum metabolites, during the progression of chronic DILI. However, there are still a few limitations in the study. As all know, due to the abundant metabolites and biological process, the metabolomic study needs to further verify the results using animal models. And multi-center, large-sample studies could improve the application of these metabolic fingerprints to clinical DILI management in the future. Nevertheless, the study is expected to offer references for clinical diagnosis and monitoring of the occurrence and development of chronic DILI-associated cirrhosis, and to serve as a resource of metabolic adaptation and reprogramming to guide future investigations on clinical prognosis, mechanisms and novel therapeutics.

## Data Availability Statement

The original contributions presented in the study are included in the article/Supplementary Material, further inquiries can be directed to the corresponding author/s.

## Ethics Statement

The studies involving human participants were reviewed and approved by Medical Ethics Committee of Fifth Medical Center of Chinese PLA General Hospital. The patients/participants provided their written informed consent to participate in this study.

## Author Contributions

J-bW, X-hX, and Z-sZ were responsible for the study concept and design. S-sC, YH, and Y-mG performed sample collection, conducted LC-MS data analysis, and manuscript preparation. S-sC and J-bW drafted the manuscript. S-sL and ZS reviewed and modified the manuscript. MN helped with data analysis. All authors contributed to the article and approved the submitted version.

## Conflict of Interest

The authors declare that the research was conducted in the absence of any commercial or financial relationships that could be construed as a potential conflict of interest.

## Supplementary Material

Supplementary Figure 1 Histological and demographic information comparison with cirrhosis and decompensation associated fingerprints. **(A,B)** Cirrhosis and decompensation associated fingerprints were positively correlated with fibrosis stage in liver biopsy. **(C,D)** Cirrhosis and decompensation associated fingerprints did not show significantly statistical difference on age and sex. S, fibrosis stage.

Supplementary Table 1 The detailed information about 30 metabolites in metabolic fingerprint for cirrhosis.

Supplementary Table 2 The detailed information about 25 metabolites in metabolic fingerprint for decompensation.

Supplementary Table 3 Metabolites whose difference may affect the clinical phenotypes on cirrhosis and decompensation.
