# Supplementary figures and images for: Serum Metabolomic Analysis of Chronic Drug-Induced Liver Injury With or Without Cirrhosis
Source: Front Med (Lausanne). 2021 Mar 29;8:640799. doi: 10.3389/fmed.2021.640799 (PMC8039323; doi:10.3389/fmed.2021.640799)

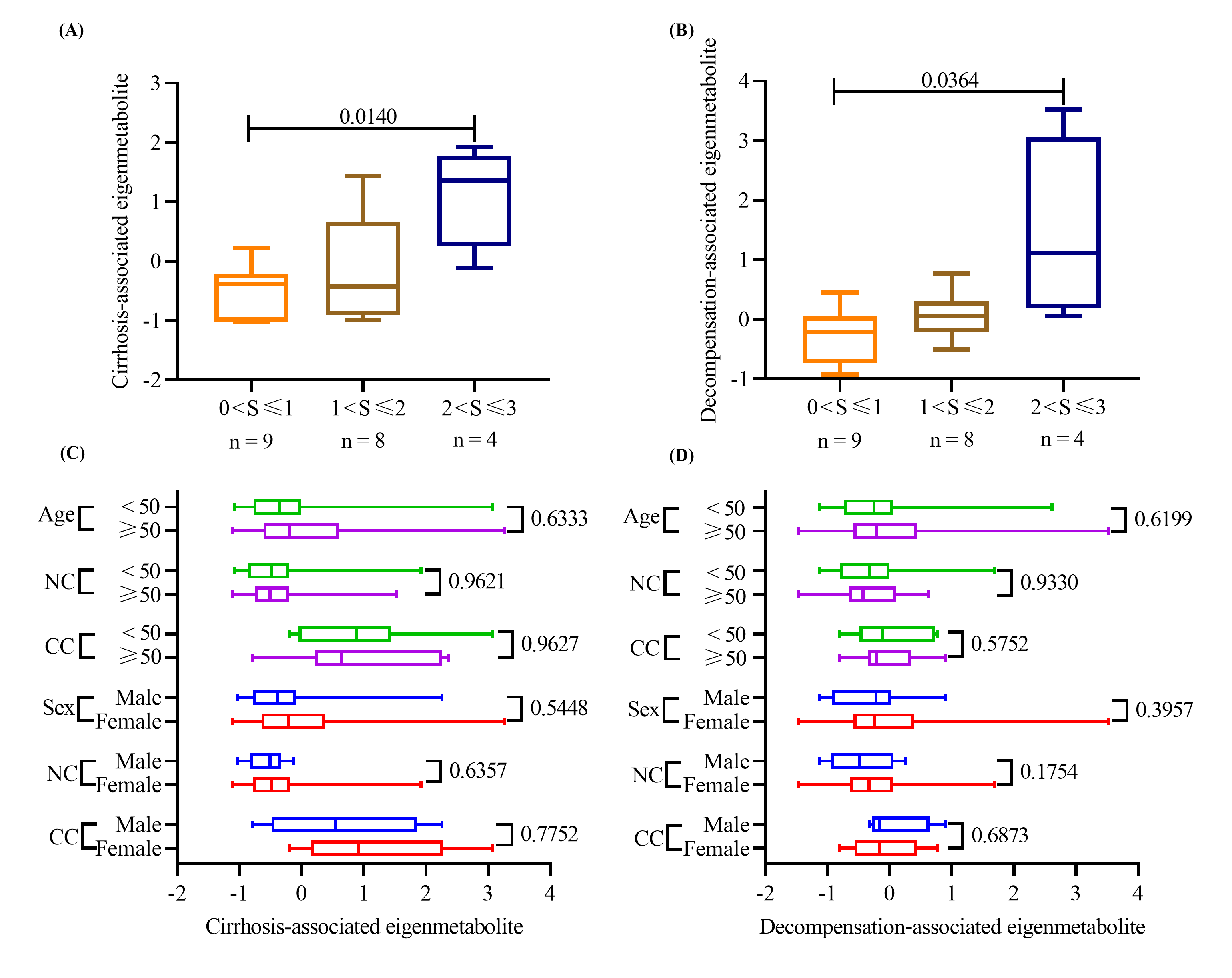

Supplement: Supplementary Figure 1 — Histological and demographic information comparison with cirrhosis and decompensation associated fingerprints. (A,B) Cirrhosis and decompensation associated fingerprints were positively correlated with fibrosis stage in liver biopsy. (C,D) Cirrhosis and decompensation associated fingerprints did not show significantly statistical difference on age and sex. S, fibrosis stage. [file Image_1.TIF]
