# Supplementary material for: Serum Metabolomic Analysis of Chronic Drug-Induced Liver Injury With or Without Cirrhosis
Source: Front Med (Lausanne). 2021 Mar 29;8:640799. doi: 10.3389/fmed.2021.640799 (PMC8039323; doi:10.3389/fmed.2021.640799)
Supplement: Supplementary Table 1 — The detailed information about 30 metabolites in metabolic fingerprint for cirrhosis. [file Table_1.DOCX]

**Table S1. The detailed information about 30 metabolites in metabolic fingerprint for cirrhosis.**

| No. | Model | MZ | RT/min | Name | HMDB | Formula | Cirrhosis/NC | | | CC/NC | | | DC/NC | | |
| --- | --- | --- | --- | --- | --- | --- | --- | --- | --- | --- | --- | --- | --- | --- | --- |
|  |  |  |  |  |  |  | AUC | FC | P value | AUC | FC | P value | AUC | FC | P value |
| 1 | ESI^-^ | 451.1365 | 3.00 | 5alpha-Androstan-3beta,17alpha-diol disulfate | HMDB0240625 | C19H32O8S2 | 0.734 | 1.962 | 0.000 | 0.750 | 1.89 | 0.000 | 0.714 | 2.053 | 0.000 |
| 2 | ESI^-^ | 255.1134 | 4.38 | 2-(3-Carboxy-3-aopropyl)-L-histidine | HMDB0011655 | C10H16N4O4 | 0.751 | 4.207 | 0.000 | 0.753 | 3.121 | 0.000 | 0.749 | 5.589 | 0.000 |
| 3 | ESI^-^ | 166.0565 | 1.22 | 3-Methoxyanthranilate | HMDB0060374 | C8H9NO3 | 0.700 | 1.709 | 0.000 | 0.634 | 1.514 | 0.012 | 0.785 | 1.957 | 0.000 |
| 4 | ESI^-^ | 498.2879 | 7.04 | Taurochenodesoxycholic acid | HMDB0000951 | C26H45NO6S | 0.771 | 4.515 | 0.000 | 0.796 | 5.281 | 0.000 | 0.738 | 3.541 | 0.009 |
| 5 | ESI^-^ | 164.0716 | 1.53 | L-Phenylalanine | HMDB0159744 | C9H11NO2 | 0.720 | 1.46 | 0.000 | 0.742 | 1.512 | 0.001 | 0.691 | 1.393 | 0.021 |
| 6 | ESI^-^ | 499.2915 | 6.97 | Tuftsin | HMDB0005770 | C21H40N8O6 | 0.697 | 4.332 | 0.001 | 0.724 | 5.534 | 0.000 | 0.663 | 2.803 | 0.036 |
| 7 | ESI^-^ | 149.0444 | 1.10 | D-Xylulose | HMDB0001644 | C5H10O5 | 0.709 | 1.514 | 0.001 | 0.625 | 1.209 | 0.172 | 0.816 | 1.903 | 0.000 |
| 8 | ESI^-^ | 191.0196 | 1.51 | Citric acid | HMDB0000094 | C6H8O7 | 0.753 | 1.801 | 0.001 | 0.724 | 1.667 | 0.029 | 0.791 | 1.971 | 0.005 |
| 9 | ESI^-^ | 320.0722 | 1.37 | Beta-Citryl-L-glutamic acid | HMDB0013220 | C11H15NO10 | 0.705 | 1.718 | 0.002 | 0.700 | 1.806 | 0.006 | 0.711 | 1.606 | 0.035 |
| 10 | ESI^-^ | 271.2269 | 14.88 | 9-Hydroxypalmitic acid | HMDB0112187 | C16H32O3 | 0.694 | 1.538 | 0.004 | 0.658 | 1.622 | 0.011 | 0.739 | 1.430 | 0.059 |
| 11 | ESI^-^ | 297.2423 | 15.00 | 3-keto stearic acid | HMDB0010736 | C18H34O3 | 0.702 | 1.571 | 0.006 | 0.615 | 1.299 | 0.258 | 0.813 | 1.917 | 0.001 |
| 12 | ESI^-^ | 464.3004 | 6.49 | Glycocholic acid | HMDB0000138 | C26H43NO6 | 0.690 | 3.72 | 0.007 | 0.678 | 4.846 | 0.002 | 0.705 | 2.287 | 0.188 |
| 13 | ESI^-^ | 826.5378 | 1.17 | PC(22:6(4Z,7Z,10Z,13Z,16Z,19Z)/18:3(9Z,12Z,15Z)) | HMDB0008732 | C48H78NO8P | 0.700 | 1.467 | 0.008 | 0.742 | 1.699 | 0.002 | 0.647 | 1.172 | 0.321 |
| 14 | ESI^-^ | 448.3056 | 6.90 | Chenodeoxycholic acid glycine conjugate | HMDB0000637 | C26H43NO5 | 0.702 | 2.488 | 0.009 | 0.72 | 2.178 | 0.032 | 0.680 | 2.883 | 0.015 |
| 15 | ESI^-^ | 73.0297 | 1.11 | Propionic acid | HMDB0000237 | C3H6O2 | 0.691 | 1.42 | 0.009 | 0.663 | 1.331 | 0.078 | 0.725 | 1.534 | 0.017 |
| 16 | ESI^+^ | 717.5615 | 17.73 | SM(d18:0/16:1(9Z)(OH)) | HMDB0013463 | C39H77N2O7P | 0.696 | 2.269 | 0.000 | 0.644 | 1.65 | 0.020 | 0.763 | 3.056 | 0.000 |
| 17 | ESI^+^ | 762.5914 | 24.60 | PC(14:0/20:0) | HMDB0007878 | C42H84NO8P | 0.712 | 1.772 | 0.000 | 0.768 | 2.134 | 0.000 | 0.641 | 1.313 | 0.145 |
| 18 | ESI^+^ | 154.0486 | 1.39 | 3-Hydroxyanthranilic acid | HMDB0001476 | C7H7NO3 | 0.712 | 2.479 | 0.000 | 0.653 | 2.403 | 0.005 | 0.786 | 2.575 | 0.000 |
| 19 | ESI^+^ | 249.22 | 13.91 | (all-Z)-8,11,14-Heptadecatrienal | HMDB0041333 | C17H28O | 0.743 | 2.018 | 0.002 | 0.717 | 1.861 | 0.026 | 0.775 | 2.218 | 0.006 |
| 20 | ESI^+^ | 191.1064 | 13.07 | N(omega)-Hydroxyarginine | HMDB0004224 | C6H14N4O3 | 0.697 | 1.647 | 0.002 | 0.733 | 1.928 | 0.000 | 0.650 | 1.29 | 0.229 |
| 21 | ESI^+^ | 460.1826 | 9.11 | 5-Methyltetrahydrofolic acid | HMDB0001396 | C20H25N7O6 | 0.713 | 3.773 | 0.005 | 0.715 | 5.081 | 0.001 | 0.710 | 2.109 | 0.003 |
| 22 | ESI^+^ | 188.1745 | 1.11 | N1-Acetylspermidine | HMDB0001276 | C9H21N3O | 0.704 | 1.994 | 0.005 | 0.694 | 1.804 | 0.046 | 0.716 | 2.236 | 0.002 |
| 23 | ESI^+^ | 204.0593 | 1.29 | Indolepyruvate | HMDB0060484 | C11H9NO3 | 0.711 | 1.310 | 0.007 | 0.694 | 1.267 | 0.065 | 0.731 | 1.363 | 0.028 |
| 24 | ESI^+^ | 175.1177 | 6.75 | L-Arginine | HMDB0000517 | C6H14N4O2 | 0.709 | 1.885 | 0.009 | 0.673 | 2.115 | 0.01 | 0.755 | 1.592 | 0.081 |
| 25 | ESI^+^ | 115.0534 | 13.81 | Dihydrouracil | HMDB0000076 | C4H6N2O2 | 0.708 | 2.401 | 0.012 | 0.693 | 1.699 | 0.133 | 0.727 | 3.293 | 0.004 |
| 26 | ESI^+^ | 333.1452 | 7.25 | Tryptophyl-Glutae | HMDB0029081 | C16H20N4O4 | 0.693 | 2.742 | 0.013 | 0.633 | 3.694 | 0.003 | 0.769 | 1.530 | 0.226 |
| 27 | ESI^+^ | 219.0823 | 1.08 | Cysteinyl-Proline | HMDB0028783 | C8H14N2O3S | 0.696 | 1.351 | 0.014 | 0.713 | 1.315 | 0.068 | 0.675 | 1.397 | 0.054 |
| 28 | ESI^+^ | 165.0543 | 1.32 | Enol-phenylpyruvate | HMDB0012225 | C9H8O3 | 0.701 | 1.186 | 0.030 | 0.709 | 1.169 | 0.112 | 0.691 | 1.208 | 0.095 |
| 29 | ESI^+^ | 160.0738 | 2.70 | Indoleacetaldehyde | HMDB0001190 | C10H9NO | 0.713 | 1.808 | 0.041 | 0.640 | 1.715 | 0.144 | 0.807 | 1.928 | 0.082 |
| 30 | ESI^+^ | 166.0713 | 1.24 | 7-Methylguanine | HMDB0000897 | C6H7N5O | 0.693 | 2.776 | 0.046 | 0.688 | 3.770 | 0.019 | 0.700 | 1.511 | 0.014 |

Abbreviation: PC, phosphatidylcholines. SM, sphingomyelin
