# Supplementary material for: Serum Metabolomic Analysis of Chronic Drug-Induced Liver Injury With or Without Cirrhosis
Source: Front Med (Lausanne). 2021 Mar 29;8:640799. doi: 10.3389/fmed.2021.640799 (PMC8039323; doi:10.3389/fmed.2021.640799)
Supplement: Supplementary Table 2 — The detailed information about 25 metabolites in metabolic fingerprint for decompensation. [file Table_2.DOCX]

**Table S2. The detailed information about 25 metabolites in metabolic fingerprint for decompensation.**

| No. | Mode | MZ | RT/min | Name | HMDB | Formula | DC/CC | | | DC/NC | | |
| --- | --- | --- | --- | --- | --- | --- | --- | --- | --- | --- | --- | --- |
|  |  |  |  |  |  |  | AUC | FC | P value | AUC | FC | P value |
| 1 | ESI^-^ | 834.5106 | 1.59 | PS(18:0/22:6(4Z,7Z,10Z,13Z,16Z,19Z)) | HMDB0010167 | C46H78NO10P | 0.851 | 0.535 | 0.002 | 0.630 | 0.610 | 0.125 |
| 2 | ESI^-^ | 197.0520 | 1.78 | 2-hydroxy-2-(4-hydroxy-3-methoxyphenyl)acetic acid | HMDB0133489 | C9H10O5 | 0.851 | 0.387 | 0.003 | 0.652 | 0.443 | 0.097 |
| 3 | ESI^-^ | 157.0528 | 4.00 | Isopropylmaleate | HMDB0012241 | C7H10O4 | 0.799 | 2.238 | 0.004 | 0.750 | 1.466 | 0.382 |
| 4 | ESI^-^ | 165.0199 | 4.85 | Benzoquinoneacetic acid | HMDB0002334 | C8H6O4 | 0.805 | 2.683 | 0.011 | 0.719 | 1.761 | 0.027 |
| 5 | ESI^-^ | 166.0175 | 4.51 | Quinolinic acid | HMDB0000232 | C7H5NO4 | 0.818 | 1.505 | 0.016 | 0.678 | 1.214 | 0.233 |
| 6 | ESI^-^ | 147.0295 | 1.09 | 2-Hydroxyglutarate | HMDB0059655 | C5H8O5 | 0.779 | 1.865 | 0.022 | 0.641 | 1.057 | 0.877 |
| 7 | ESI^-^ | 760.5763 | 1.85 | PC(14:0/20:0) | HMDB0007878 | C42H84NO8P | 0.792 | 0.446 | 0.025 | 0.647 | 0.643 | 0.195 |
| 8 | ESI^-^ | 114.0562 | 0.95 | L-Proline | HMDB0003411 | C5H9NO2 | 0.773 | 0.593 | 0.028 | 0.758 | 0.566 | 0.004 |
| 9 | ESI^-^ | 792.5304 | 18.93 | PE(22:2(13Z,16Z)/18:3(9Z,12Z,15Z)) | HMDB0009557 | C45H80NO8P | 0.779 | 2.300 | 0.031 | 0.714 | 1.863 | 0.015 |
| 10 | ESI^+^ | 767.4429 | 8.97 | PA(20:5(5Z,8Z,11Z,14Z,17Z)/22:6(4Z,7Z,10Z,13Z,16Z,19Z)) | HMDB0115221 | C45H67O8P | 0.851 | 2.335 | 0.000 | 0.628 | 1.123 | 0.604 |
| 11 | ESI^+^ | 838.6992 | 24.72 | Glucosylceramide (d18:1/26:1(17Z)) | HMDB0004976 | C50H95NO8 | 0.773 | 3.964 | 0.005 | 0.705 | 2.061 | 0.051 |
| 12 | ESI^+^ | 527.0847 | 1.20 | 3-Carboxy-1-hydroxypropylthiae diphosphate | HMDB0006744 | C16H25N4O10P2S | 0.792 | 1.419 | 0.007 | 0.741 | 1.311 | 0.004 |
| 13 | ESI^+^ | 722.3026 | 23.58 | Trypanothione disulfide | HMDB0060521 | C27H47N9O10S2 | 0.805 | 0.394 | 0.008 | 0.623 | 0.619 | 0.090 |
| 14 | ESI^+^ | 1134.7817 | 24.74 | Trihexosylceramide (d18:1/24:1(15Z)) | HMDB0004883 | C60H111NO18 | 0.773 | 0.193 | 0.008 | 0.590 | 0.370 | 0.297 |
| 15 | ESI^+^ | 551.0687 | 1.24 | UDP-L-rhamnose | HMDB0012305 | C15H24N2O16P2 | 0.779 | 1.309 | 0.008 | 0.719 | 1.215 | 0.056 |
| 16 | ESI^+^ | 724.4564 | 7.99 | 3-O-Sulfogalactosylceramide (d18:1/12:0) | HMDB0012311 | C36H69NO11S | 0.779 | 0.285 | 0.009 | 0.526 | 0.462 | 0.296 |
| 17 | ESI^+^ | 212.0536 | 13.19 | Topaquinone | HMDB0011639 | C9H9NO5 | 0.799 | 1.281 | 0.013 | 0.656 | 1.104 | 0.187 |
| 18 | ESI^+^ | 1078.4313 | 23.83 | 2-Hydroxyphytanoyl-CoA | HMDB0001295 | C41H74N7O18P3S | 0.779 | 4.704 | 0.022 | 0.680 | 1.227 | 0.810 |
| 19 | ESI^+^ | 169.9779 | 6.84 | Selenocysteine | HMDB0003288 | C3H7NO2Se | 0.799 | 3.516 | 0.022 | 0.681 | 1.909 | 0.055 |
| 20 | ESI^+^ | 799.6188 | 18.95 | SM(d18:0/22:2(13Z,16Z)(OH)) | HMDB0013467 | C45H87N2O7P | 0.779 | 2.697 | 0.024 | 0.553 | 1.051 | 0.887 |
| 21 | ESI^+^ | 937.3138 | 8.95 | Bilirubin diglucuronide | HMDB0003325 | C45H52N4O18 | 0.779 | 1.823 | 0.024 | 0.691 | 1.414 | 0.138 |
| 22 | ESI^+^ | 445.3278 | 6.75 | Menatetrenone | HMDB0030017 | C31H40O2 | 0.786 | 2.950 | 0.028 | 0.675 | 1.808 | 0.053 |
| 23 | ESI^+^ | 170.0603 | 3.56 | Phosphodimethylethanolae | HMDB0060244 | C4H12NO4P | 0.786 | 0.567 | 0.031 | 0.608 | 0.837 | 0.438 |
| 24 | ESI^+^ | 311.2024 | 10.86 | Menaquinol | HMDB0060487 | C21H26O2 | 0.799 | 4.854 | 0.033 | 0.791 | 2.638 | 0.158 |
| 25 | ESI^+^ | 291.1523 | 10.82 | 3-Polyprenyl-4,5-dihydroxybenzoate | HMDB0060380 | C17H22O4 | 0.805 | 5.993 | 0.035 | 0.583 | 0.960 | 0.950 |

Abbreviation: PS, Phosphatidylserine. PE, phosphatidylethanolamine. PA, phosphatidic acid.
