# Supplementary material for: Serum Metabolomic Analysis of Chronic Drug-Induced Liver Injury With or Without Cirrhosis
Source: Front Med (Lausanne). 2021 Mar 29;8:640799. doi: 10.3389/fmed.2021.640799 (PMC8039323; doi:10.3389/fmed.2021.640799)
Supplement: Supplementary Table 3 — Metabolites whose difference may affect the clinical phenotypes on cirrhosis and decompensation. [file Table_3.DOCX]

**Table S3. Metabolites whose difference may affect the clinical phenotypes on cirrhosis and decompensation.**

| **Metabolite** | **Description** | **Increased(↑) or decreased (↓)**  **in cirrhosis** | **cirrhosis-associated fingerprint** | **decompensation-associated fingerprint** |
| --- | --- | --- | --- | --- |
| **Related to lipids** | | | |  |
| 9-Hydroxypalmitic acid | 9-Hydroxypalmitic acid belongs to the class of organic compounds known as long-chain fatty acids. | ↑ | √ |  |
| 3-keto stearic acid | 3-Oxooctadecanoic acid, also known as 3-keto stearic acid or 3-oxostearate, belongs to the class of organic compounds known as long-chain fatty acids. | ↑ | √ |  |
| PC(22:6(4Z,7Z,10Z,13Z,16Z,19Z)/18:3(9Z,12Z,15Z)) | PC(22:6(4Z,7Z,10Z,13Z,16Z,19Z)/18:3(9Z,12Z,15Z)) is a phosphatidylcholine (PC or GPCho). | ↑ | √ |  |
| SM(d18:0/16:1(9Z)(OH)) | Sphingomyelin (d18:0/16:1(9Z)(OH)) or SM(d18:0/16:1(9Z)(OH)) is a type of sphingolipid found in animal cell membranes, especially in the membranous myelin sheath which surrounds some nerve cell axons.SMs play a role in signal transduction. | ↑ | √ |  |
| PC(14:0/20:0) | PC(14:0/20:0) is a phosphatidylcholine (PC or GPCho). | ↑ | √ |  |
| (all-Z)-8,11,14-Heptadecatrienal | (all-Z)-8,11,14-Heptadecatrienal, also known as chokegard, belongs to the class of organic compounds known as fatty aldehydes. | ↑ | √ |  |
| Propionic acid | Propionic acid is biosynthesized as propionyl CoA, which is a product of the catabolism of fatty acids having odd numbers of carbon atoms, and of the degradation of some amino acids. | ↑ | √ |  |
| PS(18:0/22:6(4Z,7Z,10Z,13Z,16Z,19Z)) | PS(18:0/22:6(4Z,7Z,10Z,13Z,16Z,19Z)) is a phosphatidylserine (PS or GPSer). | ↓ |  | √ |
| Isopropylmaleate | 2-Isopropylmaleic acid belongs to the class of organic compounds known as methyl-branched fatty acids. | ↑ |  | √ |
| PC(14:0/20:0) | PC(14:0/20:0) is a phosphatidylcholine (PC or GPCho). | ↓ |  | √ |
| PE(22:2(13Z,16Z)/18:3(9Z,12Z,15Z)) | PE(22:2(13Z,16Z)/18:3(9Z,12Z,15Z)) is a phosphatidylethanolae (PE or GPEtn). | ↑ |  | √ |
| PA(20:5(5Z,8Z,11Z,14Z,17Z)/22:6(4Z,7Z,10Z,13Z,16Z,19Z)) | PA(20:5(5Z,8Z,11Z,14Z,17Z)/22:6(4Z,7Z,10Z,13Z,16Z,19Z)) is a phosphatidic acid. | ↑ |  | √ |
| Glucosylceramide (d18:1/26:1(17Z)) | Galabiosylceramide is a non-acidic diglycosphingolipids, i.e. a sphingolipid with two or more carbohydrate moieties attached to a ceramide unit. | ↑ |  | √ |
| Trihexosylceramide (d18:1/24:1(15Z)) | Trihexosylceramide is a glycosphingolipid which contains a trisaccharide (galactose-galactose-glucose) moiety bound in glycosidic linkage to the hydroxyl group of ceramide as the polar head group. | ↓ |  | √ |
| 3-O-Sulfogalactosylceramide (d18:1/12:0) | 3-O-Sulfogalactosylceramide is an acidic, sulfated glycosphingolipid, often known as sulfatide. | ↓ |  | √ |
| SM(d18:0/22:2(13Z,16Z)(OH)) | Sphingomyelin (d18:0/22:2(13Z,16Z)(OH)) or SM(d18:0/22:2(13Z,16Z)(OH)) is a type of sphingolipid found in animal cell membranes,especially in the membranous myelin sheath which surrounds some nerve cell axons.In humans, sphingomyelin is the only membrane phospholipid not derived from glycerol. | ↑ |  | √ |
| Phosphodimethylethanolae | Phosphomonomethylethanolae, phosphodimethylethanolae and phosphocholine were weak competitive inhibitors of the cytidylyltransferase catalyzed reaction when phosphoethanolae was used as a substrate. | ↓ |  | √ |
| 2-Hydroxyphytanoyl-CoA | 2-Hydroxyphytanoyl-CoA, also known as 3S2hphy-CoA, belongs to the class of organic compounds known as long-chain fatty acyl coas. | ↑ |  | √ |
| **Related to amino acids and proteolysis** | | | |  |
| 2-(3-Carboxy-3-aopropyl)-L-histidine | 2-(3-Carboxy-3-aopropyl)-L-histidine is an unusual ao acid that results from the post-translational modification of histidine in certain proteins. | ↑ | √ |  |
| L-Phenylalanine | Phenylalanine is an essential ao acid and the precursor of the ao acid tyrosine. | ↑ | √ |  |
| Tuftsin | Tuftsin (L-threonyl-L-lysyl-L-prolyl-L-arginine) is a peptide related primarily to the immune system function. Tuftsin is a tetrapeptide (Thr-Lys-Pro-Arg) produced by enzymatic cleavage of the Fc-domain of the heavy chain of immunoglobulin G. | ↑ | √ |  |
| Beta-Citryl-L-glutamic acid | Beta-Citryl-L-glutamic acid, also known as b-citryl-L-glutamate, belongs to the class of organic compounds known as glutamic acid and derivatives. | ↑ | √ |  |
| 3-Hydroxyanthranilic acid | 3-Hydroxyanthranilic acid is an oxidation product of tryptophan metabolism. | ↑ | √ |  |
| N(omega)-Hydroxyarginine | N(omega)-Hydroxyarginine, also known as N(ω)-hydroxy-L-arginine or 6-NOHA, belongs to the class of organic compounds known as arginine and derivatives. | ↑ | √ |  |
| Indolepyruvate | Indole-3-pyruvate is a microbial metabolite, urinary indole-3-pyruvate is produced by Clostridium sporogenes and Trypanasoma brucei. | ↑ | √ |  |
| L-Arginine | Arginine is an essential ao acid that is physiologically active in the L-form. In mammals, arginine is formally classified as a semi-essential or conditionally essential amino acid, depending on the developmental stage and health status of the individual. | ↑ | √ |  |
| Tryptophyl-Glutae | Tryptophyl-Glutae is a dipeptide composed of tryptophan and glutae. It is an incomplete breakdown product of protein digestion or protein catabolism. | ↑ | √ |  |
| Cysteinyl-Proline | Cysteinyl-Proline is a dipeptide composed of cysteine and proline. | ↑ | √ |  |
| Enol-phenylpyruvate | Enol-phenylpyruvate belongs to the class of organic compounds known as phenylpyruvic acid derivatives. Phenylpyruvic acid derivatives are compounds containing a phenylpyruvic acid moiety, which consists of a phenyl group substituted at the second position by an pyruvic acid. | ↑ | √ |  |
| Indoleacetaldehyde | Indoleacetaldehyde, also known as tryptaldehyde, belongs to the class of organic compounds known as 3-alkylindoles. In humans, indoleacetaldehyde is involved in tryptophan metabolism. | ↑ | √ |  |
| Dihydrouracil | Dihydrouracil belongs to the class of organic compounds known as pyrimidones.In humans, dihydrouracil is involved in the metabolic disorder called the beta-ureidopropionase deficiency pathway. | ↑ | √ |  |
| 3-Methoxyanthranilate | 3-Methoxyanthranilate belongs to the class of organic compounds known as m-methoxybenzoic acids and derivatives. These are benzoic acids in which the hydrogen atom at position 3 of the benzene ring is replaced by a methoxy group. | ↑ | √ |  |
| 2-hydroxy-2-(4-hydroxy-3-methoxyphenyl)acetic acid | Vanillylmandelic acid, also known as vanillylmandelate or VMA, belongs to the class of organic compounds known as methoxyphenols. | ↓ |  | √ |
| Quinolinic acid | Quinolinic acid, also known as quinolinate, belongs to the class of organic compounds known as pyridinecarboxylic acids. | ↑ |  | √ |
| L-Proline | L-proline is one of the twenty ao acids used in living organisms as the building blocks of proteins. Proline is sometimes called an io acid, although the IUPAC definition of an ie requires a carbon-nitrogen double bond. Proline is a non-essential ao acid that is synthesized from glutamic acid. It is an essential component of collagen and is important for proper functioning of joints and tendons. | ↓ |  | √ |
| Selenocysteine | Selenocysteine is an ao acid that is present in several enzymes.Selenocysteine has a structure similar to cysteine, but with an atom of selenium taking the place of the usual sulfur. | ↑ |  | √ |
| Topaquinone | Topaquinone (TPQ), is the quinone of 2,4,5-trihydroxyphenylalanine. TPQ is the cofactor in most copper-containing ae oxidases. | ↑ |  | √ |
| **Related to intermediate metabolites** | | | |  |
| Citric acid | Citric acid (citrate) is a weak acid that is formed in the tricarboxylic acid cycle or that may be introduced with diet. The evaluation of plasma citric acid is scarcely used in the diagnosis of human diseases. | ↑ | √ |  |
| 3-Carboxy-1-hydroxypropylthiae diphosphate | 3-Carboxy-1-hydroxypropyl-ThPP is an intermediate in Citrate cycle (TCA cycle). 3-Carboxy-1-hydroxypropyl-ThPP is the second to last step in the synthesis of Succinyl-CoA and is converted from 2-Oxoglutarate via the enzyme 2-oxoglutarate dehydrogenase E1 component (EC.1.2.4.2). It is then converted to S-Succinyldihydrolipoamide-E via the enzyme 2-oxoglutarate dehydrogenase E1 component (EC.1.2.4.2). | ↑ |  | √ |
| Menatetrenone | Menatetrenone, also known as MK-4, is a vita K compound used as a hemostatic agent, and also as adjunctive therapy for the pain of osteoporosis. Menatetrenone is one of the nine forms of vita K2 and is a short-chain menaquinone. | ↑ |  | √ |
| Menaquinol | Menaquinol belongs to the class of organic compounds known as prenylated hydroquinones. | ↑ |  | √ |
| 3-Polyprenyl-4,5-dihydroxybenzoate | 3-Polyprenyl-4,5-dihydroxybenzoate, also known as 3,4-dihydroxy-5-polyprenylbenzoate, belongs to the class of organic compounds known as hydroxybenzoic acid derivatives. | ↓ |  | √ |
| **Related to steroids, bile acids** | | | |  |
| Taurochenodesoxycholic acid | Taurochenodesoxycholic acid is a bile acid formed in the liver by conjugation of chenodeoxycholate with taurine, usually as the sodium salt. | ↑ | √ |  |
| Glycocholic acid | Glycocholic acid is an acyl glycine and a bile acid-glycine conjugate. | ↑ | √ |  |
| Chenodeoxycholic acid glycine conjugate | Chenodeoxycholic acid glycine conjugate is an acyl glycine and a bile acid-glycine conugate.This compound usually exists as the sodium salt and acts as a detergent to solubilize fats for absorption and is itself absorbed. It is a cholagogue and choleretic. | ↑ | √ |  |
| 5alpha-Androstan-3beta,17alpha-diol disulfate | 5alpha-Androstan-3beta,17alpha-diol disulfate belongs to the class of organic compounds known as sulfated steroids. These are sterol lipids containing a sulfate group attached to the steroid skeleton. | ↑ | √ |  |
| Bilirubin diglucuronide | Bilirubin diglucuronide is a glucuronidated version of bilirubin, a tetrapyrrole compound produced via heme degradation. | ↑ |  | √ |
| **Other metabolites** | | | |  |
| **Related to vitamin metabolism** | | | |  |
| 5-Methyltetrahydrofolic acid | 5 methyltetrahydrofolic acid (5-MTHF) is the most biologically active form of the B-vita known as folic acid, also known generically as folate. 5-MTHF functions, in concert with vita B12, as a methyl-group donor involved in the conversion of the ao acid homocysteine to methionine. | ↑ | √ |  |
| **Related to purine metabolism** | | | |  |
| 7-Methylguanine | 7-Methylguanine, also known as N7-me-g, belongs to the class of organic compounds known as hypoxanthines. | ↑ | √ |  |
| **Uncategorized** | | | |  |
| D-Xylulose | D-Xylulose is a monosaccharide containing five carbon atoms. D-Xylulose is converted from xylitol by the enzyme NAD+-linked xylitol dehydrogenase (EC 1.1.1.9) in the glucuronate pathway, the most important xylitol-handling metabolic pathway in mammals. | ↑ | √ |  |
| N1-Acetylspermidine | N1-Acetylspermidine is a polyae.Aliphatic polyaes occur ubiquitously in organisms and have important functions in the stabilization of cell membranes, biosynthesis of inforg molecules, cell growth and differentiation, as well as adaptation to osmotic, ionic, pH and thermal stress. | ↑ | √ |  |
| Benzoquinoneacetic acid | Benzoquinoneacetic acid (BQA) is an oxidized form of homogentisic acid. The oxidation of homogentisic acid to BQA is catalyzed by the enzyme polyphenol oxidase. | ↑ |  | √ |
| 2-Hydroxyglutarate | 2-Hydroxyglutarate exists in 2 isomers: L-2-hydroxyglutarate acid and D-2-hydroxyglutarate.In humans it is part of butanoate metabolic pathway and can be produced by phosphoglycerate dehydrogenase (PHGDH). | ↑ |  | √ |
| UDP-L-rhamnose | UDP-L-rhamnose, also known as UDP-gal or GDU, belongs to the class of organic compounds known as pyrimidine nucleotide sugars. | ↑ |  | √ |
| Trypanothione disulfide | Trypanothione disulfide, also known as oxidized trypanothione or TSST, belongs to the class of organic compounds known as cyclic peptides. | ↓ |  | √ |
